# Hydroponics: current trends in sustainable crop production

**DOI:** 10.6026/97320630019925

**Published:** 2023-09-30

**Authors:** Ganapathy Rajaseger, Kit Lun Chan, Kay Yee Tan, Shan Ramasamy, Mar Cho Khin, Anburaj Amaladoss, Patel Kadamb Haribhai

**Affiliations:** 1 Centre for Research & Opportunities in Plant Science (CROPS), School of Applied Science, Temasek Polytechnic, 21 Tampines Ave 1, Singapore 529757; 2 Centre Centre for Aquaculture and Veterinary Science (CAVS), School of Applied Science, Temasek Polytechnic, 21 Tampines Ave 1, Singapore 529757

**Keywords:** Smart Farming, Hydroponics, IoT, Sensors, Domotics, Remote Cultivation, Data Acquisition, Future smart farming

## Abstract

The combination of Hydroponics with smart technology in farming is novel and has promise as a method for effective and environmentally friendly crop production. This technology eliminates the need for soil and reduces water usage by providing nutrients straight to the plant's roots. The Internet of Things (IoT), sensors, and automation are all used in "smart farming," which allows for constant monitoring of soil conditions, nutrient levels, and plant vitality to facilitate fine-grained management and optimization. The technology-driven strategy improves crop output, quickens growth rates, and keeps conditions ideal all year round regardless of weather or other environmental circumstances. In addition, smart farming lessens the need for organic chemical inputs, promotes environmentally safe methods of pest management, and minimizes the amount of waste produced. This ground-breaking strategy may significantly alter the agricultural sector by encouraging regionalized food production, enhancing food security, and adding to more resilient farming practices. This comprehensive review delves into current trends in Hydroponics, highlighting recent advancements in smart farming systems, such as Domotics, Data Acquisition, Remote Cultivation, and automated AI systems. The review also underscores the various types and advantages of smart farming hydroponic technology, emphasizing the requirements for achieving efficiency in this innovative domain. Additionally, it explores future goals and potential developments, paving the way for further advancements in hydroponic smart farming.

## Background:

Hydroponics is the practice of growing plants in a nutrient-rich water solution instead of soil [1]. The term "hydroponics" originated from the Greek- "hydro," which means water, and "ponos," which means labour [2]. Peat moss, charcoal, gravel, rock wool, perlite, coco peat, and coconut coir are only some of the inert media used in hydroponic systems to support plant roots [3, 4]. The system may be engineered to give the plants the optimal quantity of water, nutrients, and oxygen for optimal development. Hydroponic systems are used commercially and in private homes to cultivate a wide variety of plant life, including vegetables, fruits, herbs, and flowers. While the concept of soilless growing has been around for millennia, the modern hydroponic system was developed in the middle of the 20th century as a solution to boost food production in locations with limited resources and space [5]. It is believed that the Hanging Gardens were created utilizing the first known application of hydroponic cultivation [6]. To prove that plants might grow successfully without soil, a Flemish botanist named Jan van Helmont undertook experiments in the 17th century. Later, in 1800, French scientists De Saussure and Boussingault proved that plants require carbon, hydrogen, oxygen, and nitrogen for proper growth. Subsequently, in 1860, German scientists Sachs and Knop added to De Saussure and Boussingault's list by cultivating plants in aqueous solutions including salts of phosphorus, sulphur, potassium, calcium, and magnesium [7,8]. William Frederick Gericke is often cited as an early proponent of growing plants without soil. Because he believed it would be more efficient and successful, Gericke focused on developing methods for cultivating plants without soil. Using lettuce, tomatoes, and cucumbers as examples, he conducted a slew of experiments demonstrating that plants could grow and flourish in nutrient-rich water so long as their fundamental nutritional demands were met. The concept of soilless agriculture owes a great deal to Gericke's research, which also laid the framework for modern Hydroponics [9]. Hydroponics is now widely utilized in commercial and domestic settings to cultivate various products, from leafy greens to tuber crops to tomatoes and herbs. Its promise to boost food production and sustainability, decrease water consumption, and raise crop yields has recently increased in popularity [10].

## Importance and relevance of Hydroponics in urban farming:

According to the Food and Agriculture Organization's report in 2001, it is projected that the global population will reach 9.7 billion by 2050. This will require a 60% rise in the production of food worldwide. The current state of under nourishment has found to affect around 11% of the global population, and there has been an upward trend in this figure in recent times. As though, food security has emerged as a prominent concern in the current era, representing a critical challenge for the agricultural sector [Tilman, 2002] [11-12,13].

Furthermore, the number of malnourished individuals stands at approximately 690 million. According to research, livestock farming utilizes around 80% of the global agricultural land, necessitating significantly more land than plant-based food farming. According to research, approximately 30% of the global food supply is wasted annually [14]. According to recent data, most of the world's population resides in urban areas, surpassing the number of individuals living in rural regions. Specifically, in 2018, cities accommodated 55% of the global population [15]. According to another study, the percentage of the global population residing in urban areas was 30% in 1950 [16]. However, projections indicate that by 2050, this figure is expected to increase to 68% [17]. Even though, urban populations have been rapidly increasing, nevertheless, agriculture has been able to meet their growing demands by producing food that requires higher energy, land, water, and generates increased greenhouse gas emissions. The primary concern lies in whether agriculture can sustainably keep up with the changing demands of urban populations while also promoting agricultural prosperity and reducing poverty in both rural and urban regions [18]. When taking the perspective of urbanisation and limiting the poverty, focusing on smart farming is a far more effective way to reduce poverty than investing in other areas, as agriculture has given its pivotal role in a country's infrastructure and its potential impact on conflicts and wars which were driven by historical land-related food disputes [19]. Such that the expansion of urban areas creates competition for resources such as soil, water, and labour with agriculture. This could be attributed to the influx of people and the reclassification of land use [20]. The competition for limited resources has led to a situation where advanced agriculture is required to increase its productivity to addressing the issue on diminishing the burden of poverty chain and simultaneously combating demand of land and climate change concerns [21].

Hydroponics, however, is a crucial feature of contemporary farming, especially in the context of "smart farming." It offers some benefits and addresses serious problems with traditional agricultural methods. Hydroponics has several advantages over conventional agricultural techniques like using soil and greenhouses. Hydroponics allows for economical water consumption, typically using as much as 90% less water than conventional farming methods [22]. Hydroponics, with its carefully calibrated nutrient solutions, may produce far greater quantities of greens than conventional soil gardening [23]. Hydroponics also allows for greater crop output per unit area because of its vertical farming methodology, which makes the most efficient use of available space [24]. It was recorded that there was a significant decrease in photosynthetic photon flux density and shoot fresh weight in the Vertical Farming Systems [VFS] as the distance from the apex to the foundation increased. However, despite this reduction, the VFS generated a greater amount of crop per unit of cultivation space compared to the horizontal hydroponic system [25]. The study's findings indicate that VFS may be a viable substitute for horizontal hydroponic development mechanisms.

Additionally, the study suggests that integrating artificial lighting into the VFS may lead to even higher yields. Pests and illnesses are less likely to be a problem with hydroponic systems, lowering the demand for chemical pesticides [26]. Finally, Hydroponics allows for continuous production throughout the year independent of external weather conditions, guaranteeing a steady supply of fresh vegetables [27]. Hydroponics often provides a better Return On Investment [ROI] than conventional farming methods because of its greater productivity and quicker harvest times [28]. Research findings indicate that hydroponic lettuce production has the potential to generate significantly higher yields per acre in comparison to soil-based cultivation, with reported increases of up to 20 times. The enhanced productivity observed in hydroponic farming systems has been found to result in improved financial returns and a faster return on investment. Compared to conventional agricultural methods, hydroponic systems may increase lettuce yields by as much as 20 times per acre [29]. Brault et al., conducted a study on the year-round production of lettuce using the nutrient film technique in a greenhouse with artificial lighting to investigate the feasibility of using this technique to produce lettuce throughout the year, which involved the use of a greenhouse with artificial lighting to provide the necessary light and the nutrient film technique to provide the necessary nutrients to the plants. The study's findings indicated that hydroponic lettuce farming in a controlled environment enables uninterrupted and reliable year-round crop yield, thus surmounting any seasonal constraints [30].

By implementing a controlled environment and precise nutrient management, the authors demonstrated the feasibility of achieving uninterrupted tomato production regardless of external weather conditions, facilitating year-round market supply [31]. The researchers achieved continuous pepper production throughout the year by maintaining optimal environmental conditions, including temperature, light, and nutrient supply [32]. In another study, the research findings indicate that implementing hydroponic cultivation methods and regulated environmental factors enabled uniform strawberry yield, irrespective of seasonal constraints [33]. These benefits demonstrate the practicality and longevity of growing hydroponically-cultivated leafy greens.

## Advantages of hydroponic farming System:

Resource Efficiency: The optimization of resources like water, nutrients, and space is a key benefit of Hydroponics. Hydroponic farming techniques have been found to reduce water usage by up to 90% compared to conventional soil-based farming. Additionally, this method allows for the recycling and reutilization of nutrient solutions, promoting sustainability and minimizing waste. The implementation of vertical farming techniques has been found to optimize space utilization, resulting in the ability to achieve high-density crop production [34].

## Year-Round Crop Production:

Hydroponics allows for cultivation throughout the year without being affected by seasonal or climatic constraints. Growers can utilize controlled environments to establish the ideal conditions for plant growth, encompassing temperature, humidity, light intensity, and nutrient concentrations. Maintaining a consistent and dependable food supply, decreasing reliance on imported goods, and improving food security are all important factors to consider [35].

## Increased Crop Yields:

Research has shown that hydroponic systems accurately regulate growth conditions, leading to increased plant growth rates and greater crop yields than traditional farming techniques. Research has shown that customizing nutrient delivery and optimizing the root zone environment can improve plant health and productivity. [36].

## Environmental Sustainability:

The utilization of Hydroponics has been found to reduce the adverse effects of agriculture. The elimination of soil dependency results in a reduction of soil erosion, loss of nutrients, and spoilage. Integrating Hydroponics with sustainable practices, such as organic pest control, water recycling, and renewable energy sources, can contribute to a more environmentally friendly approach to farming [37].

## Nutritional Quality and Flavour:

The precise control of nutrient levels and growing conditions in hydroponic cultivation can improve crop quality and flavour. Hydroponic cultivation can be particularly noteworthy for speciality crops, herbs, and medicinal plants due to the potential to optimize specific compounds and active ingredients. [38].

## Pest and Disease Control:

Hydroponics provides a soil-less environment, reducing the risk of soil-borne pests and diseases. Integrated Pest Management [IPM] techniques can be implemented more effectively in Hydroponics, utilizing biological controls and minimizing the need for chemical pesticides. This leads to healthier plants and safer produce [39]. Urban Agriculture and Local Food Production: Research suggests that Hydroponics is a suitable method for urban agriculture as it allows food production in confined spaces such as rooftops, vertical farms, or indoor facilities. Promoting local food production has been found to positively reduce transportation distances and improve access to fresh, regionally produced crops. Strengthening local involvement and knowledge about sustainable food systems is also observed [14]. Scientific Research and Innovation: Hydroponics is a platform for scientific research and innovation in agriculture. Researchers can explore new techniques, develop improved cultivars, and test novel approaches for sustainable food production. Integrating smart technologies, automation, and data analytics further advances the field and facilitates continuous improvements in hydroponic farming practices. Hydroponics is a game-changer in modern agriculture, offering increased efficiency, sustainability, and productivity. Its ability to overcome the limitations of traditional farming methods makes it a valuable tool for meeting the challenges of food security, environmental conservation, and the growing demand for nutritious and high-quality produce.

## Types of Hydroponic Systems:

Hydroponic systems can be classified mainly into two based on the substrate used soilless-solution culture and granular-substrate culture Hydroponics [40]. Types of hydroponic systems that are in use today and their specification with the list of plants that grow best in them are tabulated (Table 1). 

## Overview on Recent developments in Hydroponics:

Innovative technologies, including smart home technology (domotics), IoT automated growing techniques, and AI-based systems, have increasingly entered the mainstream recently and, to add to that, have relevant applications in indoor hydroponic productions [46, 47]. The amount and accessibility of knowledge on the web means that increasing numbers of individuals are starting to explore these growth strategies for various motives, and both hydroponic and indoor cultures are growing in popularity with farmers [48].

## Domotics for indoor cultivation- control tools:

It is important to consider the concept carefully while establishing a facility for indoor production and hydroponic planting. Specifically, the spot, dimension, targeted plant species, and tools required for the particular task must be considered. A hydroponic farming system and an indoor conservatory must operate with various specialized gadgets. To provide the optimal climate for the growth of plants, we will consequently need lights with specialized ranges for horticulture, aspirators for the movement of air, humidifiers, fans, heat producers, etc. Thermo-hygrometers for determining both humidity and temperature, heating systems that regulate aspirators or cooling systems, and hygrostats to regulate humidifiers or dehumidifiers are a few control devices employed nowadays for improved and controlled production. However, advanced Software-based controls with programs that can handle information collected by sensors and regulate the operation of lights, aspirators, humidifiers, etc., through in-built applications to maintain stable every environmental condition are inadequate now.

## Data Acquisition for Cultivation:

The effectiveness or lack thereof of the yield might be influenced by a wide range of internal and external elements, including conventional methods of manual monitoring and measurements, which tend to be ineffective and insufficient in various ways. In this time when information is easily accessible, sustaining the authenticity of the agricultural system depends on reliable information gathering and dissemination. These numbers and facts are statistics compiled to provide backed-up, empirical results that might greatly enhance yield quality [49]. As a result, farming will become increasingly data-driven and data-enabled. Even though machines do a sizable portion of processing work, individuals will still be engaged. Innovative agriculture combines various automated technologies with big data availability to optimize and increase the production of crops to eliminate the need for human intervention and labour [50]. The technical developments brought about by globalization now include innovative agriculture. More automation and machine learning in farming are anticipated to result from new technologies like the Internet of things (IoT) and cloud computing that could implement data and communication processes, increasing production [51]. Large firms and companies are anticipating that massive Data in Peta and Zeta bytes has an enormous opportunity to generate revenue in various manners [52]. The term "Internet of Things" (IoT) describes an interconnected system of objects, tools, automobiles, constructions, and other technological sensing devices, like software enabling the exchanging of data. This primarily includes Radio Frequency Identification (RFID), and sensors. The fusion of the natural environment with computing devices and online resources offers valuable data and functionality for improved production [53]. In a study in 2017 to validate the effect of Internet of the Things on Smart Hydroponic Farming Ecosystem (HFE)" sensors and relays were exploited efficiently. The authors incorporated the monitoring and regulating characteristics into their prototype design to validate variables for 27 days: temperature of air and water, moisture, pH level, electrical conductivity (EC), water height, flow rate and nutrient concentration. The Arduino 2560-based information recorder was developed to gather data from five sensors on six distinct variables and store it on an SD card to show system efficiency instantaneously [54]. Kyaw et al. designed a prototype utilizing sensors to collect data and actuators to regulate settings to validate aquaponics (growing fish with Hydroponics). They employed smartphone apps for quick systems management and cloud-based storage for their regression data assessment of fish and plant growth [55].

## Remote cultivation:

Typically, a remote monitoring system comprises two overarching classifications of components, namely the remote telemetry units (RTUs) and the master stations. In broad terms, the Remote Terminal Units (RTUs) acquire data, while the master units analyze and execute commands based on the acquired information. Remote Terminal Units (RTUs) operate by being configured to collect exact categories of data. Every device is designed to oversee particular elements of the agricultural land and transmit a notification to the central system in case of any deviation from the predetermined parameters [56].

## Integration of IoT in Vertical Farming:

Vertical farming is a popular trend in Hydroponics that involves stacking multiple layers of plants in a vertical arrangement. This farming method saves space, reduces water usage, and increases yields per square foot of growing area. In response to increased demand for agricultural productivity, vertical farming is a new technology that aims to boost crop yield per unit of land. VF is a technically challenging and pricey crop production method that uses protected horticulture systems like glasshouses and controlled environment facilities along with numerous layers of growth surfaces and/or inclined production surfaces. As a result, VF requires a scientific approach that considers various elements, including lighting, crop nutrition, growing systems, energy efficiency, construction, and site selection [9]. The Internet of Things (IoT)is used in vertical farming to monitor environmental conditions and collect data on individual plants. IoT systems use this information to formulate accurate recommendations for the amounts of light, water, and nutrients that should be provided to each plant. An IoT device prototype for smart vertical farming with LED lights, sensors, a wooden board, and a battery is presented in the International Journal for Research in Applied Science and Engineering Technology. The prototype is equipped with sensors such as a light-detection resistor (LDR), soil moisture sensor (SMS), and LM35 temperature sensor (TMS), which together gather data on plant development and then analyze and show that data in a web application for optimal efficiency [57]. Recently, there have been reports of a smartphone app developed in Android Studio that allows users to regulate and track plant development in hydroponic vertical farming systems. Using Internet of Things technology, sensors are used to monitor environmental and dietary factors including temperature, humidity, TDS, pH, and water level. The Thing-Speak cloud platform was then used to send the data. The Tashi Home Pindfresh system and Arduino and Raspberry Pi have been utilized as control centres [58].

## Aeroponics Technology:

Aeroponics is an indoor horticulture technique that suspends plant roots in a nutrient-rich mist, allowing maximum oxygenation and nutrient uptake. This highly efficient farming method can result in faster plant growth and higher yields. According to Martin-Laurent et al. (1999) [10], aeroponics technology is modern, relevant, and novel. For reforestation of damaged land in humid climates, it can cultivate plants in huge quantities and tree seedlings linked to soil microorganisms, such as AM fungi.

## Aquaponics: A sustainable hydroponics approach to the circular economy:

It is the method of cultivating plants and animals [often fish] close to one another. Bacteria convert noxious fish wastes like ammonia into plant-friendly nitrates and nitrites. Aquaponics relies on fish waste as its primary food supply; thus, understanding this concept is essential. In turn, the plants filter the water, making it safe for the fish. This approach is beneficial since it helps save money and biological resources by reducing waste [59,60].

## AI-Driven Hydroponics:

Glenn Dbritto et al. have researched land and water conservation using an artificial intelligence system in the hydroponic cultivation of Tomato F1 hybrid Suhyana seed. The system provides a controlled environment where a combination of water, nutrient solution, and light is autonomously supplied to the plant roots. This approach aims to optimize plant growth while minimizing water usage and promoting sustainable land use practices [61]. A Deep neural network (DNN) was implemented in a study by Mehra et al., to regulate the hydroponic system's efficacy parameters (environmental conditions) [62]. Sensors linked to both an Arduino and a Raspberry Pi 4 have been integrated into a prototype indoor IoT-based hydroponic control system for the nutrient film technique; to automatically adjust and manage the nutrient and pH levels in the study system [63]. A recent study conducted by Sun Park involved the development and implementation of an integrated system that utilizes IoT-Edge-AI-Cloud to track environmental data in strawberry hydroponics to identify optimal harvest times. The monitoring system is suggested to gather, organize, and visualize data related to the circumstances in which strawberries are grown. Additionally, a deep learning algorithm was utilized to classify the maturity level of strawberries in images. An integrated interface was employed to visualize the monitoring and analysis results, offering fundamental data for strawberry cultivation. Authors demand that even if the area used for strawberry farming grows, the suggested system, which is based on a virtualized container and the IoT-Edge-AI-Cloud idea, may be readily scaled and flexible. The hydroponic strawberry atmosphere was monitored for 4 months to verify the effectiveness of the monitoring system. Furthermore, the verification of the harvesting was decided by utilizing strawberry images obtained from Smart Berry Farm [64].

## Factors involved in an effective hydroponic system:

## Factors affecting seed germination and seedling establishment for hydroponics system:

Regulated farming, specifically greenhouse food crop production, has been identified as a highly intensive form of cultivation that can effectively tackle the challenges of climate change, freshwater scarcity, and the increasing demand for food. The primary concern in the context of seedlings pertains to the challenge of insufficient germination and emergence. Controlling the factors that influence seed germination can improve crop development and reduce production costs by enhancing seed germination and emergence [65]. Factors influencing seed germination are tabulated in Table 2

## Effect of support system for plant growth in a hydroponic system:

Rockwool: Rockwool has been identified as a commonly used growing medium in hydroponic systems. The material under consideration is produced by melting rock and spinning it into fibres. Its notable properties include high water retention capacity and adequate aeration. The use of Rockwool has been found to provide a stable structure for root development, which in turn promotes efficient nutrient uptake. The versatility and accessibility of this substance make it suitable for various plant species [72]. Coco coir: Using coco coir as a growing medium is a sustainable and eco-friendly practice, given that it is derived from coconut husks. The material under investigation exhibits favourable water retention characteristics and facilitates adequate aeration. The use of coco coir has been found to have a positive impact on root growth, as well as creating a favourable habitat for beneficial microbial activity. The capacity to buffer nutrient solutions is a recognized characteristic of the subject, which guarantees the appropriate nutrient supply to the plants [72]. Peat moss: Peat moss is a frequently utilized growth substrate in hydroponic systems. The material exhibits exceptional water-holding properties and demonstrates a high capacity for moisture retention. Improper soil management can lead to compaction, which can impede the growth of roots. The acidity of peat moss necessitates careful monitoring and adjustment of the nutrient solution's pH. Adding perlite or vermiculite is a common practice to enhance aeration in the medium [72]. Expanded clay pellets: The lightweight and aerating properties of expanded clay pellets, also referred to as hydroton or clay pebbles, are making them highly interested in the hydroponics field now. Additionally, these pellets have been noted to provide effective drainage. They exhibit a pH value of 7, indicating a neutral nature. Furthermore, it displays a notable resistance to degradation and does not undergo breakdown over extended periods. Using clay pellets is prevalent in hydroponic systems that operate through flood and drain (ebb and flow) mechanisms. Plant roots require stability and efficient oxygen exchange, which specific structures can facilitate. The water-holding capacity of these materials is limited, which may necessitate more frequent irrigation [72]. Sponge: Sponges and foam cubes have been identified as common support systems utilized in aeroponic or nutrient film technique (NFT) hydroponic systems. Providing a stable structure for seed germination and root development is crucial to plant growth and development. According to studies, sponges exhibit notable water retention capabilities and can retain nutrient solutions near roots. They also facilitate adequate aeration and prevention of waterlogging. The maintenance of sponges may necessitate meticulous observation to avoid desiccation or excessive saturation. Biochar: Biochar production involves subjecting organic matter to high temperatures in an oxygen-free environment, producing carbon-rich material with a porous structure. The material exhibits outstanding characteristics of water retention and aeration. Research has shown that applying biochar can enhance the capacity of the root zone to retain nutrients and promote microbial activity. Moisture regulation is one of the benefits of this technique, which also mitigates the risk of overwatering. The low nutrient-holding capacity of the soil may necessitate supplementary fertilization [73,74]. Classification of Hydroponics based on the usage of substrates in the medium is listed in Table 3.

## Effect of Nutrients in Hydroponics and its Importance:

Plant growth primarily depends on the availability of 17 essential nutrients. Which in turn can be broadly classified into macronutrients and micronutrients? The importance of both for the nourishment and growth of plants cannot be overstated. This includes macro-nutrients like carbon, hydrogen, oxygen, nitrogen, phosphorus, potassium, sulphur, calcium, and magnesium and micro-nutrients like iron, manganese, zinc, boron, molybdenum, chlorine, copper, and nickel. Plants acquire carbon, hydrogen, and oxygen through natural means, specifically from the air and water they consume, with the remainder obtained from the soil. The roots obtain nutrients from nutrient solutions or aggregate media in hydroponic systems. Research has shown that hydroponic systems are comparatively less tolerant than soil-based systems, and any issues related to nutrients can rapidly manifest plant symptoms. The criticality of the nutrient solution composition and regular monitoring of the nutrient solution and plant nutrient status is a significant aspect to consider. The major salt deficiencies that a hydroponic system may encounter include nitrogen, calcium, iron, magnesium, and boron deficiencies. The detrimental effects of soluble salts have been attributed to various factors, including but not limited to over-fertilization, suboptimal water quality, gradual accumulation of salts in the aggregate media, and inadequate drainage. Insufficient leaching during the process of fertigation in Hydroponics can lead to the accumulation of soluble salts in the medium due to water evaporation [75]. Nutrient antagonism and interaction is a crucial parameter that warrants serious consideration in the context of hydroponic systems. Research suggests that plants tend to absorb nutrients proportionally to their presence in the nutrient solution. The phenomenon of nutrient uptake in excess leading to a higher uptake of one nutrient at the cost of yet another has been observed and is classified as nutrient antagonism. The nutrient levels in the nutrient solution may not necessarily guarantee optimal plant growth and development. Despite sufficient nutrient supply, plant nutrient deficiency may still occur [75,76]. Some major nutrients significance has been discussed below.

## Nitrogen deficiency:

In instances of nitrogen deficiency, the colour of leaves may change to a lighter shade of green or, in severe cases, a yellow hue. Observations can be made from stunted development and discolouration, specifically a slight purple tint, on the stems and undersides of leaves. Whereas if the feeding solution includes excessive nitrogen, roots become stunted, causing blossoming to be delayed [77].

## Boron Deficiency:

Boron deficits are rare and often accompany calcium shortages, mainly in the case of plant's deficit with water. Boron generally improves root uptake of potassium and phosphorus and keeps plant cell walls intact and functioning [78]. Xin Song and colleagues studied hydroponic sugar beetroot growth and yield to determine the impact of Boron (B) depletion. Sugar beetroot seedlings' SLW and root-shoot ratio increased after exposure to a Boron deficit for 14 days. Enzyme activities such as peroxidase, catalase, and superoxide dismutase have decreased due to this deficiency, whereas malonaldehyde and proline have increased. Oxidative stress was caused by the accumulation of ROS in plant cells and the subsequent loss of antioxidant enzyme performance. According to this research, a lack of boron can negatively impact a plant's development and structure [79].

## Magnesium Deficiency:

Hydroponic production requires a full hydroponic nutrient solution, which includes Mg as one of the key ingredients. Mg insufficiency can be made worse by nutritional inconsistencies. Magnesium (Mg) uptake can be blocked by certain ions, such as Ca and K. Higher levels of either Ca or K can inhibit Mg uptake; therefore, the standard goal ratio is 2:1 for Ca:Mg and 4:1 for K:Mg. Mg deficiency can also be caused by a few other, less frequent factors, including a cool root zone or a stunted root system due to illness or waterlogging. As Mg is poorly accessible at low pH (e.g., pH less than 5), a low substrate pH can also lead to Mg insufficiency [80]. However, in their study on cormrbid conditions of Mg deficiency in mulberry plants, Rajesh Kumar Tewari et al., has implicated the induction of oxidative stress and antioxidant responses in these plants due to Mg deficiency in a hydroponic condition oxidative stress and antioxidant responses in mulberry plants. They have also reported a significant decrease in H2O2 production in Mg deficit plants.

## Iron Deficiency:

An alkaline soil growing system similar to that found in sugar beet fields was created by buffering the hydroponic medium with sodium bicarbonate (NaHCO3) to characterize iron deficiency. The study claimed that the in vivo ability for Fe3+-chelate decrease boosted substantially in both Fe efficient genotypes (NB1 and NB1xNB4) but less than two times in the Fe inefficient genotype (NB4). It was found that the distribution and period of enhanced Fe3+-chelate decline capacity were contingent on the Fe efficiency integrity of individual genetic makeup [81]. In another study, Low Fe levels influence pigment and micronutrient contents of chile pepper (*Capsicum annuum* L.) were studied through a hydroponic system. It was found that the total extractable pigments of red fruits and their surface colour remained unaffected by iron treatment. However, leaf Fe and Fe ++ were directly proportional to iron supplement, on the other hand, indirectly proportional to copper, phosphorus, and zinc concentrations in the leaf [82].

## pH level of nutrient solution:

pH indicates the solution's acidity or alkalinity. 0-14, with 7 neutrals. Maintaining nutrition solution pH levels in the optimal range increases nutrient availability. Soilless culture nutrient solutions should have a pH between 5 and 7 (typically 5.5) as they are weakly neutralized and needs automated pH adjustment to keep the root environment between 6 and 6.5 [83]. Even though Phosphorous (H2PO4 to HPO4) buffers pH, pH between 1 to 10 mM, it is harmful to plants. A circulating solution with around 0.05 mM has substantially less buffering power than the new replenishment mixture that replaces transpiration losses because plants actively absorb phosphorus [84]. Nutrients commonly employed in nutrient solutions of hydroponic growing systems, their functions, and the diseases associated with their deficiency in plants are tabulated in Table 4.

## Effect of physical factors:

## Light:

The significance of light in photosynthesis cannot be overstated, as it is the primary source of energy for plants to synthesize organic compounds. Through this process, plants convert light energy into chemical energy, which is then utilized to support their metabolic processes and promote growth. Artificial lighting systems such as LED lights can be utilized in smart Hydroponics to regulate light's intensity, spectrum, and duration meticulously. The optimization of light settings is a crucial factor in plant growth, as it enables growers to provide the appropriate amount and quality of light that is required for each growth stage. The light requirements of plants vary depending on the species, with some requiring specific amounts of red and blue light. Light regulation in smart Hydroponics is crucial for providing plants with adequate energy for photosynthesis, which leads to healthy growth, strong development, and enhanced yield [85].

## Temperature:

Temperature plays a significant role in plant growth and metabolic processes. In smart Hydroponics, the temperature can be precisely regulated to create an ideal plant environment. Each plant species has an optimal temperature range for growth and development, including germination, root growth, and flowering. Maintaining the appropriate temperature range can enhance enzymatic activity, nutrient uptake, and overall plant performance. Smart hydroponics systems often use sensors and automated controls to monitor and adjust temperature levels, ensuring that plants are kept within their preferred temperature range [85].

## Humidity:

The term humidity pertains to the quantity of water vapour in the atmosphere. The careful management of humidity in smart Hydroponics can lead to the creation of an optimal growing environment. The impact of high humidity levels on transpiration rates in plants has been studied, with findings suggesting potential benefits for certain plant species during the vegetative growth stage. Research has shown that high humidity levels can lead to the development of fungal diseases. Low humidity has been found to cause rapid moisture loss in plants, potentially resulting in water stress. Incorporating humidifiers, dehumidifiers, or ventilation systems in smart hydroponics systems enables the maintenance of accurate humidity levels. The manipulation of humidity levels by growers can facilitate an optimal environment for plants, fostering robust growth and mitigating the likelihood of pathogenic infections [86]. In smart Hydroponics, the ability to control and optimize physical factors gives growers greater precision and flexibility in creating an ideal growing environment. By fine-tuning light, temperature, and humidity, growers can mimic optimal conditions for specific plant species, growth stages, and environmental preferences. This level of control allows for more efficient resource utilization, improved plant health, and, ultimately higher yields in hydroponic cultivation.

## Advantages of hydroponic smart farming:

## Increased Yield:

Precise control over environmental factors promotes optimal plant growth. Nutrient-rich solutions lead to healthier and more vigorous plants and higher yields than traditional soil-based cultivation methods.

## Water Efficiency:

Hydroponics uses up to 90% less water than soil-based farming. Recirculating systems minimize water wastage and evaporation. Water is efficiently delivered directly to plant roots, reducing water usage.

## Space Efficiency:

Hydroponic systems are highly space-efficient and require less land. Vertical growing techniques maximize production in limited areas. Suitable for urban farming, rooftops, or areas with limited agricultural space.

## Controlled Environment:

Precise control over light, temperature, humidity, and nutrient levels is needed. Ideal growing conditions tailored to specific plant requirements. Year-round cultivation regardless of seasonal limitations.

## Reduced Environmental Impact:

Less land and water usage minimize the ecological footprint. Decreased need for pesticides and herbicides. Minimized soil erosion and nutrient runoff, preserving soil quality.

## Superior Plant Quality:

Enhanced nutrient delivery promotes healthy plant growth. Higher concentrations of desired compounds in herbs and medicinal plants. Improved flavour, aroma, and nutritional value.

## Rapid Growth and Harvest:

Plants grow faster in Hydroponics due to optimized growing conditions. Shorter crop cycles and faster harvest times. Quick turnaround and increased production capacity.

## Disease and Pest Control:

Soil-free environment minimizes the risk of soil-borne diseases and pests. Easier monitoring and management of plant health. Reduced reliance on chemical treatments.

## Sustainability:

Efficient resource utilization reduces waste and promotes sustainability. Water and nutrient recycling systems minimize environmental impact. Lower carbon footprint compared to traditional farming methods.

## Flexibility and Scalability:

Hydroponic systems can be scaled up or down to suit different needs. Versatile setups accommodate various plant species-adaptability to different growing environments and locations. By harnessing these advantages, Hydroponics could offer a highly efficient and sustainable method of cultivation, enabling growers to maximize yields, optimize plant quality, and minimize environmental impact.

## Future Goal:

Achieving 30% food sustainability by 2030 with only 1% arable land requires careful planning, innovation, and the implementation of various strategies. Some key considerations for achieving this goal include:

## Hydroponics in hospitals:

Integrating mobile hydroponic systems in hospitals is a potential future direction for Hydroponics. This approach is considered innovative and can improve nutrition and patient care. Culturing high-value plants rich in essential nutrients, minerals, vitamins, and therapeutic compounds can improve the quality of meals in hospitals. This targeted nourishment may support patients' recovery and overall well-being. Integrating a mobile hydroponic system with vertical farming techniques can optimize cultivation in a small area, allowing for efficient use of space in various settings such as roof-top gardens, balconies, or dedicated indoor spaces. Implementing this approach guarantees an uninterrupted provision of superior plant specimens such as herbs, leafy greens, or medicinal plants. Using these plants in cooking, teas, or extracts can serve as a natural source of remedies and supplements for patients, which may aid in fulfilling their dietary needs. Hydroponics provides a controlled environment that enables year-round cultivation, reducing reliance on seasonal produce and potential supply fluctuations.

## Vertical Farming and Rooftop Gardens:

Vertical farming and rooftop gardens should be extensively explored due to the limited availability of land. Vertical farming involves growing crops in vertically stacked layers, enabling optimal utilization of space. Establishing rooftop gardens on buildings has been identified as a potential method for expanding areas available for food production. [87].

## The practice of indoor agriculture:

It encompasses greenhouse systems, can facilitate crop production throughout the year while safeguarding crops from unfavourable weather conditions. The practice of Controlled Environment Agriculture (CEA) entails meticulously regulating environmental variables, including light, temperature, humidity, and CO2 concentrations, to facilitate the most favourable conditions for plant growth. Implementing CEA technologies has potentially improved productivity and resource efficiency [88].

## High-Yield Crop Selection:

Hydroponic system studies emphasize the importance of prioritizing high-yield crops that can provide maximum output within a confined space. The prioritization of crops with shorter growth cycles, higher nutritional value, and greater demand is a crucial aspect of agricultural research. This study aims to investigate and determine appropriate crop cultivars that are well-suited for the climatic conditions of Singapore and can be cultivated effectively through hydroponic or other advanced agricultural methods.

## Efficient Resource Utilization:

The optimization of resource utilization can be achieved by implementing smart irrigation systems, water recycling, and nutrient management strategies. Implementing techniques such as drip irrigation, fogging, or precision fertigation can reduce water and nutrient wastage. Research has shown that the implementation of recirculating hydroponic systems can result in a significant reduction in water consumption.

## Agro-technology and Automation:

Implementing advanced agro-technology and automation can enhance productivity and decrease labour demands in the agricultural sector. The implementation of IoT-based systems, sensors, and data analytics are being researched to monitor and control environmental parameters, detect crop health issues, and optimize resource usage. Implementing automated processes, such as robotic seeding, harvesting, and maintenance, has the potential to address labour limitations [89]. Sustainable Energy Sources: The implementation of advanced agro-technology and automation has been shown to impact productivity and labour requirements in agriculture positively. Implementing IoT-based systems, sensors, and data analytics can be utilized to monitor and control environmental parameters, detect crop health issues, and optimize resource usage. Implementing automated processes, such as robotic seeding, harvesting, and maintenance, can potentially address labour limitations [90, 91]. Collaboration and Partnerships: Promoting collaboration among government agencies, research institutions, industry stakeholders, and the community is essential. Promoting knowledge sharing, research collaborations, and public-private partnerships is essential in driving innovation, exchanging best practices, and collectively working towards achieving food sustainability goals [92]. Education and Awareness: This study aims to increase public awareness and knowledge regarding food sustainability and the advantages of locally cultivated produce. Research suggests that urban farming initiatives in schools, community centres, and residential areas can involve citizens in sustainable food production and create a sense of food security [93]. Policy Support and Incentives: Research suggests that implementing supportive policies and incentives can effectively promote urban farming initiatives. Research suggests that providing grants, subsidies, tax incentives, and streamlined regulatory processes can effectively encourage investment in urban agriculture. The study aims to investigate the impact of policies on fostering innovation, research, and the adoption of sustainable farming practices [94]. Food Waste Management: The effective management of food waste is a crucial area of research that requires attention. The promotion of composting, recycling, and utilising food waste as a valuable resource for bioenergy or fertilizers is recommended. The reduction of food waste has the potential to alleviate the burden on resources and foster a more sustainable food system [95,96]. By considering these factors and implementing a comprehensive approach that encompasses technology, innovation, collaboration, and sustainable practices, locals can work towards achieving their 30% food sustainability goal by 2030, despite limited arable land availability.

## Conclusion:

In conclusion, Hydroponics holds immense promise for the future of agriculture. This innovative cultivation method offers a range of advantages that address the challenges faced by traditional farming practices. With its ability to maximize resource efficiency, enable year-round crop production, and enhance yields, Hydroponics has the potential to revolutionize the way we grow food. By utilizing Hydroponics, we can optimize water, nutrients, and space, reducing waste and promoting sustainability. The controlled environments of hydroponic systems allow for precise control over growing conditions, resulting in accelerated growth rates and higher crop yields. This, in turn, contributes to food security, reduces dependence on imports, and increases the availability of fresh, locally-grown produce. Moreover, Hydroponics offers a pathway to environmental sustainability by reducing soil erosion, minimizing chemical inputs, and integrating with eco-friendly pest control methods. It also opens up possibilities for urban agriculture, allowing for food production in limited spaces and bringing farming closer to urban centres. As technology advances, integrating smart technologies, automation, and data analytics with Hydroponics further enhances its potential. This integration enables real-time monitoring, precise control, and automation of various processes, leading to greater efficiency, reduced labour requirements, and improve overall productivity.

## Figures and Tables

**Table 1:** Types of Hydroponic systems and specific plants

**S.No**	**Hydroponic System**	**Characteristics**	**Specific Plants**	**Reference**
1.	Nutrient Film Technique (NFT)	The roots of plants in NFT systems are constantly bathed in a thin coating of nutrient-rich water, from which they may draw the nutrients they need. The excess liquid is collected and subjected to a recycling process	Leafy Greens, Herbs, Strawberries	[41]
			Beans	
			Raddish, Cucumber	
2.	Deep Water Culture (DWC):	This involves the suspension of plant roots in a nutrient solution that is continuously oxygenated through an air pump. The roots of plants that remain submerged in water can absorb more nutrients and oxygen due to their prolonged exposure to the aquatic environment. The Dutch bucket system is a well-liked deep-water hydroponic system that offers a productive and regulated environment for plant growth. Individual buckets or containers filled with an inert medium, such as perlite or coconut coir, support the plants while enabling their roots to reach a nutrient-rich solution. Due to its scalability, simplicity of use, and adaptability for various plants, this system is commonly employed in commercial Hydroponics.	Lettuce, Herbs, Leafy Greens, And Plants With Larger Root Systems Like Tomatoes, Cucumbers, And Peppers.	[32,40]
3.	Drip System:	The utilization of drip systems entails the direct delivery of nutrient solutions to the root zone of plants through an arrangement of tubes and drippers. The method involves dripping the solution onto the chosen growing medium, such as perlite or coco coir, and subsequently allowing it to drain back into the container for potential reuse.	Vegetables, Fruits, Herbs, And Flowering Plants.	[10]
4.	Ebb and Flow (Flood and Drain)	This involves the cyclic submersion of plants in a nutrient solution, followed by subsequent drainage back into a reservoir. The regular repetition of this cycle facilitates the provision of nutrients and oxygen to the roots.	Tomatoes, cucumbers, peppers, Strawberries. Lettuce.	[10]
			Spinach.	
			Radishes.	
			Beans.	
5.	Aeroponic	This involves the suspension of plant roots in the air, with intermittent nutrient solution application as a fine mist or spray to the roots. The observed phenomenon enables a higher level of oxygenation and nutrient uptake.	Leafy Greens, Herbs, Strawberries	[42]
			Capsicum,	
			Cauliflower,	
			Chilli	
6.	Wick System	Passive hydroponic setups known as wick systems are characterized by their simplicity. In this experimental setup, the plants are situated within an inert growing medium, and a wick composed of a cotton rope is employed to facilitate the upward movement of the nutrient solution from a reservoir to the root zone.	Small Plants, Herbs, And Low-Nutrient-Demanding Plants	[43]
7.	Krakty method	Among the other types of hydroponic methods, this system is much easier and cheaper; it does not require electronic devices and does not need electric current to operate. The entire crop needs an initial administration of water and nutrients, making the system an effective plant production method. The static, suspended-pot method is highly efficient for minimizing water wastage.	Leafy Greens, Herbs	[44] [45]

**Table 2:** Factors affecting Seed germination

**Factors**	**Effect on Seed Germination**
Temperature:	The germination of seeds is dependent on specific temperature conditions. The germination of plant species is influenced by the temperature range that is most suitable for their growth. Research has shown that ensuring appropriate temperature conditions can enhance the speed and consistency of seed germination. Maintaining the appropriate temperature for specific seeds during germination in a hydroponic system is crucial to monitoring [66].
Light:	The importance of light in the process of seed germination and early seedling development has been widely recognized in the field of plant research. It has been observed that the germination process of most seeds is triggered by exposure to light, while some seeds may require a period of darkness to initiate germination. The optimal intensity and duration of light are crucial factors in hydroponic systems. Artificial grow lights, such as LED or fluorescent lights, can provide the seedlings with the required light energy [66,67].
Water and Moisture	Hydroponic systems rely on water as the primary medium for delivering plant nutrients. Adequate water availability and moisture levels are critical for seed germination. Ensuring the seeds are consistently moist but not overly saturated is important, as excess water can lead to rotting or damping-off diseases. Maintaining an appropriate moisture balance in the growing medium is vital for successful seedling establishment. Wetting the chosen medium before planting the seeds is recommended without soaking it excessively. In order to achieve successful germination, it is crucial to maintain a consistent moisture level for the seeds. However, it is important to note that excessive water can impede the growth of the initial shoots and roots, ultimately hindering the establishment of the plant. It is also recommended to maintain a consistently moist environment for the medium during the germination process and the initial days following sprouting. Overwatering must be avoided to prevent medium saturation [68].
Oxygen	Oxygen availability is a crucial aspect that affects seed germination and root development in hydroponic systems. The respiration process of seeds necessitates oxygen, and oxygen in the growth medium is a critical factor in ensuring the successful establishment of healthy seedlings. Using an inert growing medium, such as perlite, vermiculite, or rockwool, is common in hydroponic systems. This method facilitates optimal oxygen exchange. Adequate oxygen supply to the roots is crucial to nutrient solution aeration. [69,70].
Nutrients:	Although seeds possess certain nutrients to facilitate their initial growth, further nutrients are necessary for the healthy development of seedlings. A balanced nutrient solution is required in hydroponic systems, which should contain essential macronutrients such as nitrogen, phosphorus, and potassium, along with micronutrients like iron, zinc, and manganese, in appropriate concentrations. Optimal seedling growth can be supported by ensuring a proper nutrient balance and maintaining the desired pH level of the nutrient solution [70].
pH Level:	The measurement of acidity or alkalinity in a solution is commonly referred to as pH. Plants' optimal growth depends on their specific pH requirements, which vary among plant species. Maintaining an appropriate pH range within the nutrient solution is crucial for successful germination and establishment of seedlings in a hydroponic system. Research has shown that the optimal pH range for most plants is slightly acidic to neutral, with a range of approximately 5.5 to 6.5. Frequent monitoring and adjustment of the pH level in the nutrient solution is crucial in establishing an ideal growth environment for the seeds [70].
Seed Quality:	The role of seed quality and viability is crucial in determining the success of germination. Using premium seeds from trustworthy sources enhances the probability of prosperous germination and seedling establishment. In order to maximize the germination potential of seeds, it is important to use fresh, disease-free, and properly stored seeds [71].

**Table 3:** Hydroponic system based on substrate employed as a medium

**Hydroponic System**	**Growing Medium**	**Advantages**	**Disadvantages**
**Solution Culture**			
Nutrient Film Technique	No solid medium, a thin film of nutrient solution flows over the roots	Water and nutrient efficiency	System failure risks
		Oxygenation of roots	pH and nutrient balance maintenance
		Rapid plant growth	Limited root support
		Space efficiency	Sensitivity to temperature fluctuations
		Reduced pests and diseases	Initial setup and cost
Deep Water Culture	No solid medium, roots suspended in nutrient solution	Increased oxygenation	Equipment and setup cost
		High growth rates	Monitoring and maintenance
		Water efficiency	Power dependency
		Nutrient control	Root diseases
		Reduced pests and diseases	
Aeroponics	No solid medium, roots suspended in air, misted with nutrient solution	Water and nutrient efficiency	System complexity and maintenance
		Oxygenation of roots	Vulnerability to power outages
		Rapid plant growth	Sensitivity to environmental factors
		Space efficiency	Initial setup and cost
		Reduced pests and diseases	Risk of root drying
**Medium Culture**			
Rockwool	Rockwool fibres provide support and moisture retention	Excellent water retention	Initial high cost of the material
		Good aeration for root development	Requires proper pH adjustment
		pH stability for optimal nutrient uptake	Limited availability in some regions
		Insulation against temperature fluctuations	Environmental impact due to its production process
		Disease-resistant and sterile medium	
		Reusable multiple times	
		Suitable for a wide range of plant species	
Perlite	Lightweight and porous material allowing good aeration	Aeration	Dust and floating particles
		Lightweight	Limited cation exchange capacity
		pH-neutral	Fragile and breakdown over time
		Reusability	Limited structural support
Vermiculite	Mineral-based medium with moisture retention and drainage properties	Water retention	Potential for root rot
		Aeration	Cost and availability
		Insulation	Limited reusability
		Nutrient Retention	Limited structural support
		pH-neutral	Compaction
Coco Coir	Derived from coconut husks, it provides water retention and aeration.	Aeration	High cost of the material
		Lightweight	Limited cation exchange capacity
		pH-neutral	Fragile and breakdown over time
		Reusability	Limited structural support
Expanded Clay Pebbles	Lightweight clay pebbles for aeration and support	Excellent drainage properties	High initial cost compared to other media
		Lightweight and easy to handle	Requires regular cleaning to prevent clogging
		Provides good aeration for root development	Limited water-holding capacity may require more frequent watering
		pH-neutral and inert material	It can be dusty and require rinsing before use
		Reusable multiple times	It may require additional support structures for heavy plants
		Suitable for a variety of plant species	

**Table 4:** Nutrients commonly used in hydroponic nutrient solutions

**Nutrient**	**Classification**	**Function in Plant**	**Major Source**	**Deficiency Symptoms**
Nitrogen (N)	Macro	Essential for growth, leaf development, and protein synthesis	Nitrate (NO3-) and Ammonium ( NH4+)	Stunted growth, yellowing leaves
Phosphorus (P)	Macro	Key role in root development, energy transfer, and flower/fruit formation	Phosphate (PO4-)	Poor root growth, purplish leaves
Potassium (K)	Macro	Vital for overall plant health, nutrient uptake, and disease resistance	Potassium (K+)	Weak stems, yellowing edges of leaves
Calcium (Ca)	Macro	Essential for cell wall structure and overall plant stability	Calcium (Ca2+)	Blossom end rot, stunted growth
Magnesium (Mg)	Macro	Crucial for chlorophyll production and enzyme activation	Magnesium (Mg2+)	Yellowing between leaf veins
Sulfur ( S)	Macro	Important for protein synthesis and overall plant vigor	Sulfate (SO4-)	Yellowing of younger leaves
Iron (Fe)	Micro	Required for chlorophyll formation and enzyme functions	Iron (Fe2+ and Fe3+)	Yellowing leaves with green veins
Manganese (Mn)	Micro	Necessary for photosynthesis and enzyme activities	Manganese (Mn2+)	Yellowing between leaf veins
Zinc (Zn)	Micro	Essential for enzyme activation and hormone regulation	Zinc (Zn2+)	Stunted growth, malformed leaves
Copper (Cu)	Micro	Important for enzyme functions and photosynthesis	Copper (Cu2+)	Wilting, leaf discoloration
Molybdenum (Mo)	Micro	Required for nitrogen fixation and enzyme activity	Molybdate (MoO4-)	Yellowing of older leaves
Boron (B)	Micro	Vital for cell division, pollen formation, and sugar transport	Borate (BO3-)	Brittle and distorted leaves
Nickel (Ni)		Plays a role in nitrogen metabolism and enzyme function	Nickel (Ni2+)	Reduced growth, leaf deformation
